# Interaction between silicon and organic matter improves mineral nutrition and production of sour passion fruit in a semi-arid region of Brazil

**DOI:** 10.3389/fpls.2026.1835730

**Published:** 2026-05-25

**Authors:** Denise da Silva Ramos, Evandro Franklin de Mesquita, Antônio Gustavo de Luna Souto, Caio da Silva Sousa, José Paulo Costa Diniz, Dalila Regina Mota de Melo, José Félix de Brito Neto, Alberto Soares de Melo, José Francismar de Medeiros, Reynaldo Teodoro de Fatima, Ítalo Herbert Lucena Cavalcante, Luiz Fernando de Sousa Antunes, Rennan Fernandes Pereira

**Affiliations:** 1 Postgraduate Program in Agricultural Sciences, State University of Paraíba, Campina Grande, PB, Brazil; 2 Postgraduate Program in Agronomy, Federal University of Paraíba, Areia, PB, Brazil; 3 Department of Agriculture, Federal Institute of Education, Science and Technology of Paraíba, Catolé do Rocha, PB, Brazil; 4 Postgraduate Program in Soil and Water Management, Federal University of the Semi-Arid, Mossoró, RN, Brazil; 5 Postgraduate Program in Plant Production, Federal University of the São Francisco Valley, Petrolina, PE, Brazil

**Keywords:** cattle manure, nutritional status, *Passiflora edulis* Sims, silicate fertilization, yield

## Abstract

**Introduction:**

Water restriction and low soil fertility under semi-arid conditions, particularly in sandy soils prone to nutrient imbalance and sodium accumulation, limit the yield and fruit quality of sour passion fruit. The application of silicone (Si) and organic matter (OM) has been proposed as a strategy to mitigate these constraints by improving plant nutrition and ionic balance. This study evaluated the effects of Si and cattle manure on the nutritional status, yield, and fruit quality of sour passion fruit.

**Methods:**

A field experiment was conducted in a randomized block design in a 5 × 2 factorial scheme, consisting of five Si doses (0, 27, 54, 81, and 108 g plant^-1^) combined with the presence or absence of cattle manure, with four replications and four plants per plot. Leaf macro- and micronutrients, sodium content, fruit yield, titratable acidity, and soluble solids were evaluated.

**Results:**

A significant interaction between Si doses and cattle manure was observed, with the combined application enhancing plant nutrition and fruit production. Silicon increased leaf concentrations of P, Ca, B, Mo, and Si while reducing Na accumulation, whereas cattle manure enhanced leaf N, K, Mg, S, and Fe contents, although it also increased Na levels. In addition, Si improved fruit quality by increasing titratable acidity and soluble solids. The highest fruit yield (15.69 kg plant^-1^) was obtained with 108 g plant^-1^ of Si in the presence of cattle manure. However, the dose of 81 g plant^-1^ combined with cattle manure provided the best overall agronomic performance, ensuring a more balanced improvement in nutrient status, yield, and fruit quality.

**Discussion:**

Overall, the integrated use of Si and cattle management is an effective strategy to improve nutrient uptake, ionic balance, and the performance of sour passion fruit cultivated under sandy semi-arid conditions. combined with cattle manure provided the best overall agronomic performance, ensuring a more balanced improvement in nutrient status, yield, and fruit quality.

## 1 Introduction

The production of sour passion fruit (*Passiflora edulis* Sims) is mainly concentrated in the semi-arid region of northeastern Brazil, which accounts for approximately 70% of the national output, reaching around 480,000 tons annually (IBGE, 2025). In this region, irrigation is indispensable to obtain consistent high yields and to secure farm-level profitability. However, prolonged droughts, high temperatures and elevated evapotranspiration markedly restrict soil water availability throughout most of the year (Paiva et al., 2025).

This semi-arid region is characterized by harsh climatic conditions, with mean annual precipitation typically below 800 mm and maximum temperatures that can reach 45 °C during dry periods (Araújo, 2023; Figueredo et al., 2024). Soils are commonly shallow and sandy to sandy-loam, with low organic matter content (OM) and limited native fertility (Ferreira et al., 2020; Medeiros et al., 2022). Collectively, these edaphoclimatic constraints reduce soil water retention and nutrient buffering capacity, increasing crop vulnerability to drought and saline irrigation water.

Among the strategies to mitigate abiotic stress in crops, the application of silicon (Si) has gained increasing attention. Although not considered an essential element, this beneficial element is widely recognized for enhancing plant tolerance to environmental stresses by improving structural integrity, antioxidant capacity, and membrane stability (Costa et al., 2016; Rajput et al., 2021; Queiroz et al., 2025). In addition, silicon contributes to greater nutrient-use efficiency, particularly for nitrogen and potassium, resulting in improved plant growth and yield (Silva et al., 2025). Mechanistically, these benefits are associated with stimulation of root development, increased transporter activity, and enhanced nutrient uptake and translocation within the plant (Ali et al., 2020; Wang et al., 2021; Yan et al., 2021; Narula and Chaudhry, 2025).

Concomitantly, low soil organic matter is a major limitation in the Brazilian semi-arid region due to pedogenetic processes, erosion, and intensive land use (Santos et al., 2023). This fraction plays a key role in nutrient retention, soil structure, water infiltration, and microbial activity, and its depletion reduces soil resilience to drought and salinity (Kabato et al., 2025; Marzouk et al., 2025). Organic amendments, especially cattle manure, are widely recommended to restore organic matter, improve soil physical and chemical properties, and sustain crop nutrition under marginal conditions (Medeiros et al., 2022).

Recent studies indicate that the combined use of silicon and organic amendments can promote synergistic effects on plant nutrition, stress tolerance, and fruit quality (Kang et al., 2022; Paiva et al., 2025). However, field-based evidence linking their application to changes in soil mineral dynamics and plant nutritional status under semi-arid conditions remains limited.

Continuous cropping without proper nutrient replenishment accelerates the depletion of soil organic reserves, as nutrients are removed during harvest, reinforcing the need for restorative soil management practices (Xia et al., 2025). In this context, cattle manure represents an accessible and cost-effective source of nutrients and organic inputs, improving soil fertility, aggregation, water dynamics, and biological activity.

Therefore, the combined application of silicon-based fertilization and organic amendments emerges as a promising strategy to mitigate water stress, enhance nutrient availability, and improve crop performance in semi-arid environments, with potential applicability in other water-limited agricultural systems.

We hypothesized that the combined soil application of silicon and cattle manure increases nutrient availability and improves plant nutrition, yield, and fruit quality of sour passion fruit under semi-arid conditions. Accordingly, this study aimed to evaluate the effects of soil-applied silicon in combination with cattle manure on plant nutrition, yield, and fruit quality of sour passion fruit in a semi-arid region of Brazil.

## 2 Materials and methods

### 2.1 Experimental site description

**Figure 1:**
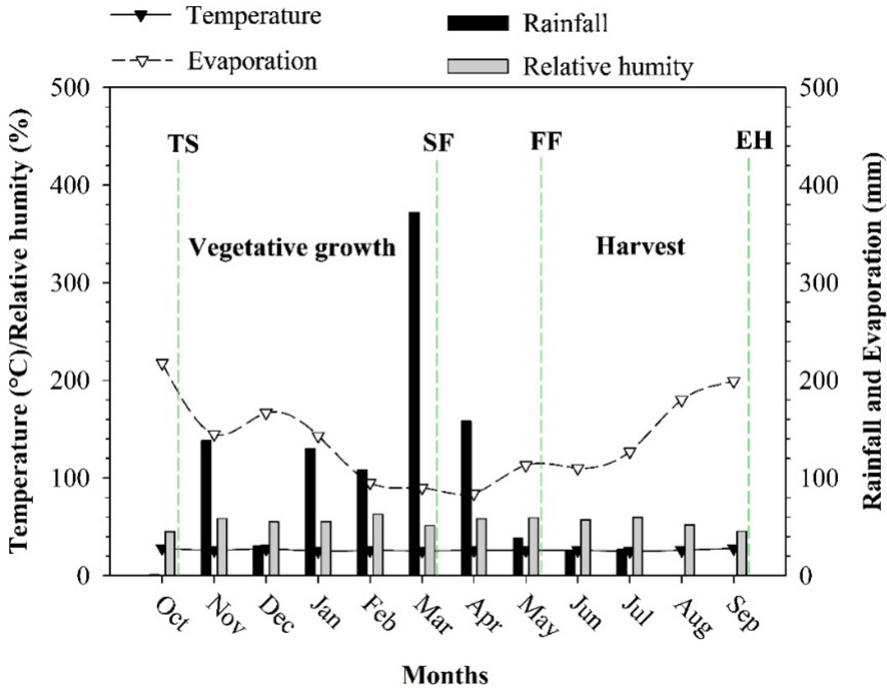
Monthly climatic data recorded during the experimental period. TS, Transplanting the seedlings; SF, Start of flowering; FF, Fruit formation; EH, End of the harvest.

The experiment was carried out from October 2022 to September 2023 in an experimental area located at the Center for Human and Agrarian Sciences, belonging to the State University of Paraíba, municipality of Catolé do Rocha, State of Paraíba, Brazil. The experimental area is georeferenced by the coordinates: latitude 6° 20’ 38” S, longitude 37° 44’ 48” W of the Greenwich Meridian and at an altitude of 275 m. The climate of the region is BSw’h’, according to Köppen’s classification, which is characterized as a hot semi-arid climate, with two distinct seasons, one rainy with irregular precipitation and the other without precipitation (Alvares et al., 2013). Mean air temperature, relative humidity, reference evaporation, rainfall, and irrigation are presented in **Figure 1**.

The soil of the experimental area, according to the criteria of the Soil Taxonomy, was classified as Entisol (Fluvent) (Soil Survey Staff, 2022). Before setting up the experiment, single soil samples were collected from the 0-0.20 m layer, homogenized and transformed into a composite sample for characterization of the chemical attributes regarding fertility and physical attributes (**Table 1**) (Teixeira et al., 2017).

**Table 1:** Characterization of the soil for chemical properties regarding fertility and physical properties before the experiment.

Fertility	
pH	6.00
P (mg dm^-3^)	16.63
K^+^ (cmolc dm^-3^)	0.08
Ca^2+^ (cmol_c_ dm^-3^)	1.09
Mg^2+^ (cmol_c_ dm^-3^)	1.12
Na^+^ (cmol_c_ dm^-3^)	0.05
SB^a^ (cmol_c_ dm^-3^)	2.34
H^+^+Al^3+^ (cmol_c_ dm^-3^)	1.24
Al^3+^ (cmol_c_ dm^-3^)	0
CEC^b^ (cmol_c_ dm^-3^)	3.58
V^c^ (%)	65.36
OM^d^ (g kg^-1^)	13.58
Physical properties	
Sand (g kg^-1^)	831.5
Silt (g kg^-1^)	100.0
Clay (g kg^-1^)	68.5
WDC^e^ (g (kg^-1^)	0.00
DF^f^ (kg dm^-3^)	1
BD^g^ (g cm^-3^)	1.53
PD^h^ (g cm^-3^)	2.61
TP^i^(m^3^ m^-3^)	0.42
H0.03MPa^j^ (MPa)	49
H1.50MPa^l^ (MPa)	28
Textural class	Loamy sand

^a^ SB, Sum of exchangeable bases (SB = Ca^2+^+Mg^2+^+K^+^+Na^+^); ^b^CEC, Cation exchange capacity [CEC, SB (Ca^2+^+Mg^2+^+K^+^+Na^+^)]; ^c^V, Soil saturation by exchangeable bases [V, (SB/CEC) × 100; OM, Organic matter; ^e^WDC, Water-dispersible clay; ^f^DF, Degree of flocculation {DF, [(WDC-Clay)/Clay] × 100}; ^g^BD and ^h^PD, bulk and particle density, respectively; ^i^TP; ^j^H0.03MPa, ^l^H1.5MPa, Respectively, volumetric moisture contents at field capacity and permanent wilting point at soil tensions of -0.033 and -1.500 MPa.

### 2.2 Experimental design and plant material

The experiment was distributed in randomized blocks, in a 5 × 2 factorial scheme, referring to five doses of silicon and application of cattle manure (with and without), with four blocks and four plants per plot, considering the two central plants as the usable plot. Treatments were related to doses of 0.0, 27, 54, 81 and 108 g plant^-1^ of silicon in soil without and with application of organic matter in the form of cattle manure to raise the initial content from 1.2% (**Table 1**) to 4.0%. Silicon doses were applied to the soil prior to seedling transplanting (basal application) and then every 45 days until peak fruiting, at 225 days after transplanting (DAT), totaling six applications. The silicon source used was Sifol^®^, containing orthosilicic acid [Si(OH)_4_], with a chemical composition of 92% SiO_2_ and 42.9% Si, an apparent density of 80–140 g L^-1^, particle size of 8-12 µm, and pH ranging from 6.0 to 7.5.

The plant material under study was the sour passion fruit accession “Guinezinho”, propagated via seeds in a nursery accredited by the Ministry of Agriculture, Livestock and Food Supply (MAPA). Seedlings were prepared in black polyethylene bags with capacity of 1.5 L containing substrate consisting of a mixture of equal parts of topsoil material (0-0.2 m) and aged cattle manure.

Two seeds were sown in each bag, and germination started seven days after sowing (DAS) and stabilized at 30 DAS, when the plants were thinned using scissors, leaving only the most vigorous.

### 2.3 Crop management

At 60 DAS, the seedlings were taken to the field for transplanting. The training system used was the trellis with smooth wire n° 12 on top of the stakes at 2 m height and spaced 3 m apart. At the ends of each row, posts with 0.2 m diameter were fixed to withstand the tension imposed by the trellis system and the plants. The spacing used was 3 m between plants and 2 m between rows, equivalent to a density of 1667 plant ha-1. The pruning of the sour passion fruit tree consisted of: pruning of the main stem - which consisted of pruning the apical meristem of plants that reached the top of the espalier and exceeded 0.10 m (approximately 2.10 m) to stimulate lateral shoots (secondary branches) in opposite directions (east and west); pruning of secondary branches - which consists of pruning the meristem of lateral branches (secondary branches) after exceeding 1.5 m of growth in each direction of the plant (east and west) to stimulate productive branches (tertiary and quaternary); pruning of productive branches - the productive branches were pruned to 0.30 m above the soil surface to prevent the branches and fruits from coming into direct contact with the soil and causing problems with burning and diseases; cleaning pruning - consisted of removing excess, broken or diseased branches to improve aeration and phytosanitary management.

The irrigation water used in the experiment presented the following chemical characteristics: electrical conductivity of 1.01 dS m^-1^, pH of 6.9, Ca^2+^ concentration of 2.5 mmol(c) L^-1^, Mg^2+^ of 1.48 mmol(c) L^-1^, Na^+^ of 6.45 mmol(c) L^-1^, Cl^-^ of 8.1 mmol(c) L^-1^, HCO_3_^-^ of 2.75 mmol(c) L^-1^, SO_4_^2-^ of 0.18 mmol(c) L^-1^, and a sodium adsorption ratio (SAR) of 4.57 (mmol L^-1^)^1/2^.

The irrigation method used was drip irrigation with two pressure-compensating drippers with flow rate of 10 L h^-1^ installed at 0.2 m from the plant collar on opposite sides, working at a service pressure of 1.5 MPa. The daily irrigation depth was calculated by the methodology of crop evapotranspiration (ETc), which was estimated by the product between reference evapotranspiration (ET_o_) and crop coefficient (Kc), according to methodologies contained in Valipour (Valipour, 2017) and Anwar et al. (Anwar et al., 2021)– (Equation 1).

(1) $$\text{ETc}={\text{ET}}_{0}\times \text{kc}$$

The crop coefficients for sour passion fruit were 0.3 in the first 60 DAT, 0.8 from 61 to 110 DAT (beginning of flowering), 1.2 from the beginning of flowering to fruit formation (160 DAT), and 0.8 from the beginning to the end of fruit harvest (Souza et al., 2018). Near the experimental area, a class “A” pan was installed to determine ET0, by multiplying the daily evaporation (ETa) by the adjustment coefficient (Kt) of 0.75 (Equation 2).

(2) $$\text{ETo}={\text{ET}}_{\text{a}}\times {\text{K}}_{\text{t}}$$

Planting holes measuring 0.4 m × 0.4 m × 0.4 m were dug, totaling a volume of 64 dm³. As the holes were dug, the soil volume of the first 0.2 m was separated, and for those treatments with cattle manure application, 7.7 kg per hole was added to increase the initial soil content (1.2%) to 4%, calculated according to the methodology described by Bertino et al.(Bertino et al., 2015) – Equation 3, with 95 g plant^-1^ of single superphosphate - SS (21% P_2_O_5_, 16% Ca and 10% S) and 50 g plant^-1^ of FTE-BR12 (3.9% S, 1.8% B, 0.85% Cu, 2.0% Mn and 9.0% Zn).

(3) QCM=(DOMC−SOMC)×HV×BD×UCM/OMCCM

Where:

QCM = Quantity of cattle manure to be applied (kg hole^-1^);
DOMC = Desired organic matter content (g kg^-1^);
SOMC = Soil organic matter content (g kg^-1^);
HV = Hole volume (dm^3^);
BD = Bulk density (g dm^-3^);
UCM = Moisture content of cattle manure (%);
OMCCM = Organic matter content in cattle manure (%).

Prior to application, cattle manure was chemically characterized for fertility attributes (Teixeira et al., 2017), as presented in **Table 2**.

**Table 2:** Chemical characterization of cattle manure used in the experiment.

Attributes	Values
pH (H_2_O)	7.7
EC (dS m^-1^)	6.09
OM (dag kg^-1^)	36.2
OC^a^ (g kg^-1^)	166.9
N^b^ (g kg^-1^)	13.9
C/N^c^	12.0
P^d^ (g kg^-1^)	3.2
K^+e^ (g kg^-1^)	18.7
Ca^2+h^ (g kg^-1^)	16.2
Mg^2+i^ (g kg^-1^)	6.1
S^j^ (g kg^-1^)	2.5
CEC^q^(mmol dm^-3^)	133.9
B^l^ (mg kg^-1^)	14.8
Fe^m^ (mg kg^-1^)	11,1129.9
Cu^n^ (mg kg^-1^)	19.3
Mn° (mg kg^-1^)	491.4
Zn^p^ (mg kg^-1^)	65.3
Si (g kg^-1^)	12.5
Na^+f^ (g kg^-1^)	3.5

^a^ OC, Carbon oxidation by potassium dichromate and determination by colorimetry; ^b^N, Kjeldahl by dry digestion; ^c^C/N, carbon:nitrogen ratio; ^d^P, Mehlich-1 and photocolorimetry, at 660 nm; ^e^K^+^ and ^f^Na^+^, Flame photometry; ^h^Ca^2+^ and ^i^Mg^2+^, atomic absorption spectrometry at 422.7 and 285.2 nm; ^j^S – atomic absorption spectrometry at 420 nm; ^l^B and ^m^Fe, UV-vis spectrometry at wavelengths of 460 and 508 nm, respectively; ^n^Cu, atomic absorption spectrometry at 324.7 nm; °Mn and ^p^Zn, atomic absorption spectrometry at 231.9 and 279.5 nm, respectively, with acetylene air flame; ^q^CEC, cation exchange capacity.

From 30 DAT onwards, topdressing fertilization was carried out monthly, applying nitrogen (N) and potassium (K) in a 1:1 ratio and using urea (45% N) and potassium sulfate (53% K_2_O and 15% S) as sources, respectively. The fertilization of sour passion fruit, respectively for N and K, followed the recommendation proposed by Souto et al. (2023) and was carried out with 15 and 20 g until the end of the vegetative stage, 24 and 30 g from the end of the vegetative stage to the beginning of flowering, and 33 and 60 g from the end of flowering until the end of the harvest, totaling 231 and 350 g plant^-1^ year^-1^ of urea and potassium sulfate, respectively. Phosphate fertilization was carried out every three months and from 60 DAT, applying 50 g of plant^-1^ SS of single superphosphate from vegetative growth to the beginning of flowering and 100 g of SS from the end of flowering to the end of harvest, totaling 250 g plant^-1^ year^-1^ of single superphosphate.

### 2.4 Experimental analyzes

At 210 DAT, in the phenological stage of full flowering, eight leaves were collected from the median part in the two central plants of the plot of each treatment, collecting the fourth or fifth pair of leaves counted from the apex of the flowering branches, following the leaf collection criteria for nutritional analysis of sour passion fruit (Natale and Rosane, 2018). These leaves were placing into a paper bag and transported to the laboratory. After washing in distilled water, the leaves were dried in an air circulation oven at 60 °C until reaching constant weight, ground in a stainless-steel knife mill (Wiley type) and stored in an identified and hermetically sealed container.

Contents of macronutrients (N, P, K, Ca, Mg and S), micronutrients (B, Fe, Zn, Cu and Mn), sodium (Na) and silicon (Si) were determined to evaluate the nutritional status of passion fruit, using the methodologies compiled by Silva et al (Silva, 2009). Nutritional status was determined as follows: nitrogen (N) by the Kjeldahl method (dry digestion); phosphorus (P) by molybdenum blue spectrometry; potassium (K) and sodium (Na) by atomic emission spectrometry; calcium (Ca), magnesium (Mg), sulfur (S), iron (Fe) and copper (Cu) using an atomic absorption spectrometer at wavelengths of 422.7, 285.2, 400.0, 508.0 and 3,274.7 nm, respectively; boron (B) by UV-vis spectrometry at the wavelength of 460.0 nm; and zinc (Zn) by air-acetylene flame atomic absorption spectrometry. Si was determined by plasma emission spectrometry (dry digestion). The silicon (Si) content in leaf tissues was determined following a dry digestion procedure. Briefly, plant samples were oven-dried at 65 °C until constant weight and ground to a fine powder. Approximately 0.1–0.2 g of the dried material was subjected to dry ashing in a muffle furnace at 500–550 °C. After cooling, the ash was dissolved in hydrochloric acid, and the volume was adjusted with deionized water. Silicon concentration was then quantified using inductively coupled plasma optical emission spectrometry (ICP-OES), following the methodology described by Korndörfer (2004), with minor adaptations. This method was chosen due to its accuracy and suitability for Si determination in plant tissues.

Fruit harvesting was carried out daily, starting at 180 DAT, when the fruit peel color becomes predominantly yellowish, which occurs approximately 60 days after flower opening (anthesis) (Vianna-Silva et al., 2010). Harvested fruits were counted and weighed on a semi-analytical scale to calculate the production per plant (kg plant^-1^).

Fruits were harvested daily from 180 DAT, corresponding to the stage when the peel turned predominantly yellow, approximately 60 days after (anthesis)38. The number and total fresh weight of harvested fruits were recorded using a semi-analytical balance, and yield was expressed as kg plant^-1^.

The pulp of fruits was manually extracted and used for physicochemical analyzes. Soluble solids (SS) content was determined using a portable digital refractometer (Atago^®^, model PAL-1), and results were expressed in °Brix. Titratable acidity (TA) was determined according to the AOAC (2016) methodology (Equation 4). Aliquots of 5 mL of the sample were diluted in 25 mL of distilled water, followed by the addition of two drops of 1% phenolphthalein and titration with 0.1 N NaOH until a persistent light pink color appeared for 30 s. TA was calculated as follows:

(4) $$\text{TA}=\frac{\left[\text{V}\times \text{N}\times \text{f}\times 100\right]}{\text{P}}$$

Where: V is the NaOH volume (mL), N is the normality, and V_a_ is the sample volume (mL). Results were expressed as g citric acid 100 mL^-1^ of pulp.

### 2.5 Statistical analyzes

The Shapiro-Wilk test assessed the normality of the error, and the Bartlett test assessed the homogeneity of the variance, both with p > 0.05. Thus, the data demonstrated that the distribution is normal and that the variation is uniform (p > 0.05). Next, analysis of variance (Factorial ANOVA) was used with the F test (p ≤ 0.05), depending on the significance of the factors, first- and second-degree linear regression (R² > 0.60), based on the significance of the curve for the silicon factor and the interaction, and the F test (p ≤ 0.05) for the organic matter factor. Data analysis, principal component analysis (PCA) was performed using the packages available in R 4.5.1 (R Core Team, 2021).

## 3 Results

According to factorial ANOVA, there was a significant effect of the silicon doses × organic matter interaction for phosphorus, calcium, boron, molybdenum and silicon leaf contents and fruit production of sour passion fruit (**Table 3**). Leaf contents of nitrogen, potassium, magnesium, iron, manganese, zinc, solids soluble, and titratable acidity responded to the individual application of silicon and organic matter (p ≤0.05). Si application had a significant effect on the leaf contents of copper and sodium, while organic matter had a significant effect on the leaf sulfur content of sour passion fruit.

**Table 3:** Summary of the analysis of variance, by the mean square value, for the leaf contents of macronutrients, micronutrients, sodium, silicon, postharvest quality, and fruit production of sour passion fruit under doses of Si, silicon and application of OM, organic matter.

SV^a^	Block	Si	OM	Si × OM	Residual	Total	CV^c^
DF^b^	3	4	1	4	27	39	
N	6.88*	5.55*	222.38**	0.64^ns^	2.07	–	5.38
P	2.82^ns^	4.17**	32.13**	5.79**	0.033	–	10.05
K	1.25^ns^	5.68**	41.91**	0.16^ns^	5.20	–	13.22
Ca	1.98^ns^	9.00**	0.64^ns^	3.19*	3.08	–	13.83
Mg	1.55^ns^	10.19**	8.05**	1.93^ns^	0.030	–	10.01
S	2.71^ns^	2.24^ns^	106.19**	0.18^ns^	0.026	–	6.64
B	0.51^ns^	7.17**	11.97**	3.79*	71.95	–	18.57
Fe	8.35**	32.10**	44.11**	1.39^ns^	9.60	–	3.60
Mn	2.64^ns^	4.74*	114.96**	1.73^ns^	68.38	–	11.84
Cu	1.84^ns^	5.47**	3.41^ns^	1.58^ns^	29.49	–	24.07
Zn	17.60**	10.07**	5.21*	1.69^ns^	2.15	–	5.66
Mo	0.45^ns^	13.12**	100.50**	10.21**	0.18	–	23.06
Na	0.49	5.18**	7.58	1.40^ns^	337562.53	–	15.18
Si	8.23**	54.87**	4.12*	6.71**	159257.49	–	12.46
Prod	3.23^ns^	68.47**	6.29^ns^	29.61**	2.56	–	14.62
SS	0.22^ns^	7.74**	0.29^ns^	1.20^ns^	1.38	–	8.73
TA	0.32^ns^	3.63*	0.76^ns^	1.01^ns^	0.35	–	12.81

^a^ SV, Source of variation; ^b^DF, Degrees of freedom; ^c^CV, coefficient of variation; Prod, fruit production; SS, soluble solids; TA, titratable acidity; *, ** and ^ns^, significant at 5% probability level, 1% probability level and not significant, respectively, by the F test.

**Figure 2:**
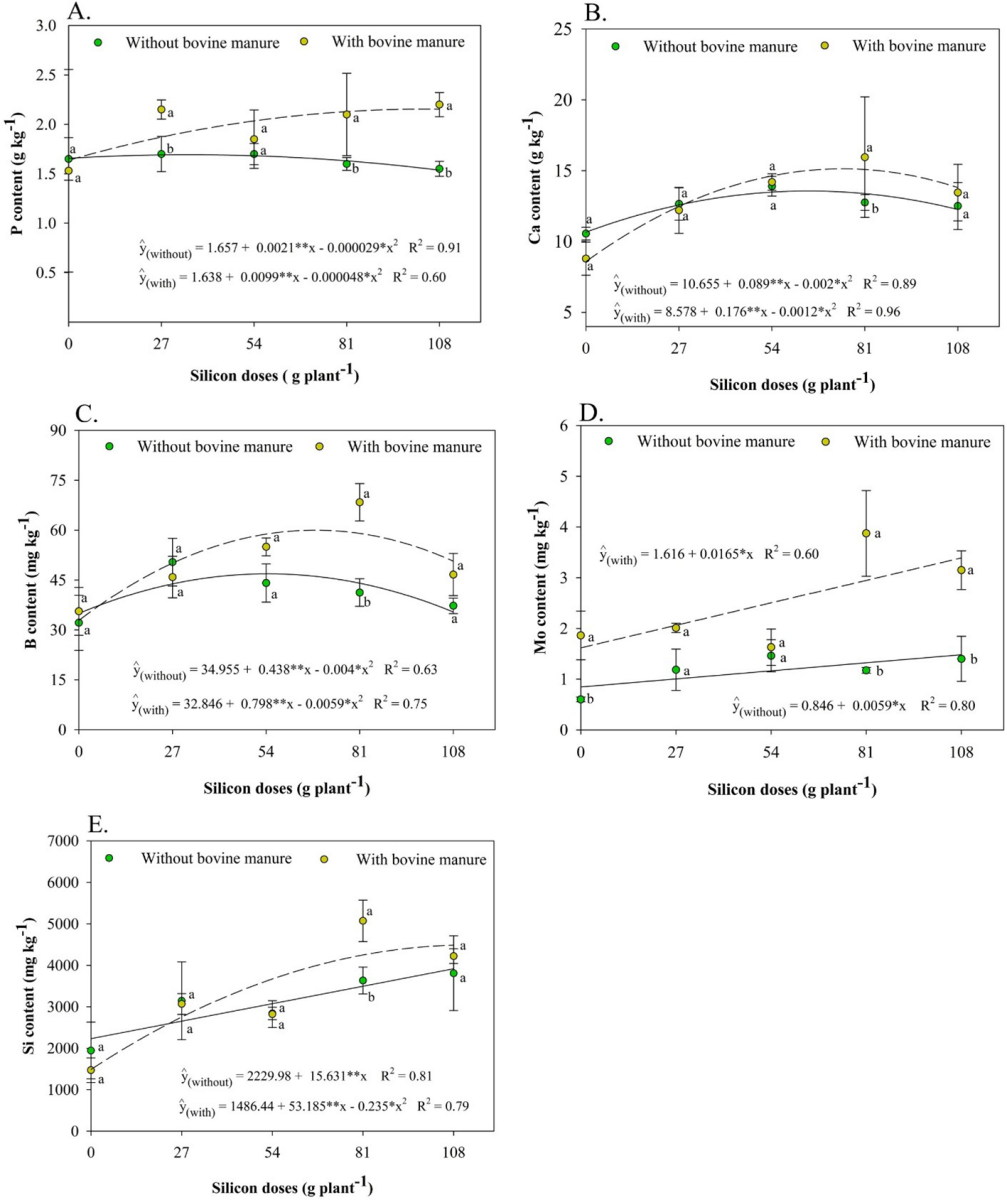
Leaf contents of phosphorus **(A)**, calcium **(B)**, boron **(C)**, molybdenum **(D)** and silicon **(E)** in sour passion fruit as a function of the application of silicon and organic matter doses in the soil. **,*Values significant at 1% and 5% probability by the F test, respectively. Error bars represent the standard error of the mean (n = 4).

The application of silicon in the soil, especially when associated with organic matter, increases the leaf contents of P, Ca, B, Mo and Si in sour passion fruit (**Figure 2**). The dose of 108 g plant^-1^ of silicon applied together with cattle manure increased the leaf contents of P, Mo and Si, respectively, from 1.63 to 2.14 g kg^-1^ (**Figure 2A**), 1.61 to 3.34 mg kg^-1^ (**Figure 2D**) and 1486.44 to 3445.74 mg kg^-1^ (**Figure 2E**). At the highest dose of Si applied, cattle manure promoted increments of 104.78% in leaf P content and 271.11% in leaf Mo content in sour passion fruit.

Leaf Ca contents were increased as Si doses increased up to 63.57 g plant^-1^ (without manure) and 73.33 g plant^-1^ (with manure), with maximum estimated values of 13.39 and 15.03 g kg^-1^, respectively (**Figure 2B**). Similarly, for leaf B contents, the values were increased up to 46.94 and 59.82 mg kg^-1^, respectively, at the doses of 54.75 g plant^-1^ (without manure) and 67.62 g plant^-1^ (with manure), as shown in **Figure 2C**. It was observed that organic matter promoted increments of 12.24% in Ca content and 27.43% in Mo content.

**Figure 3:**
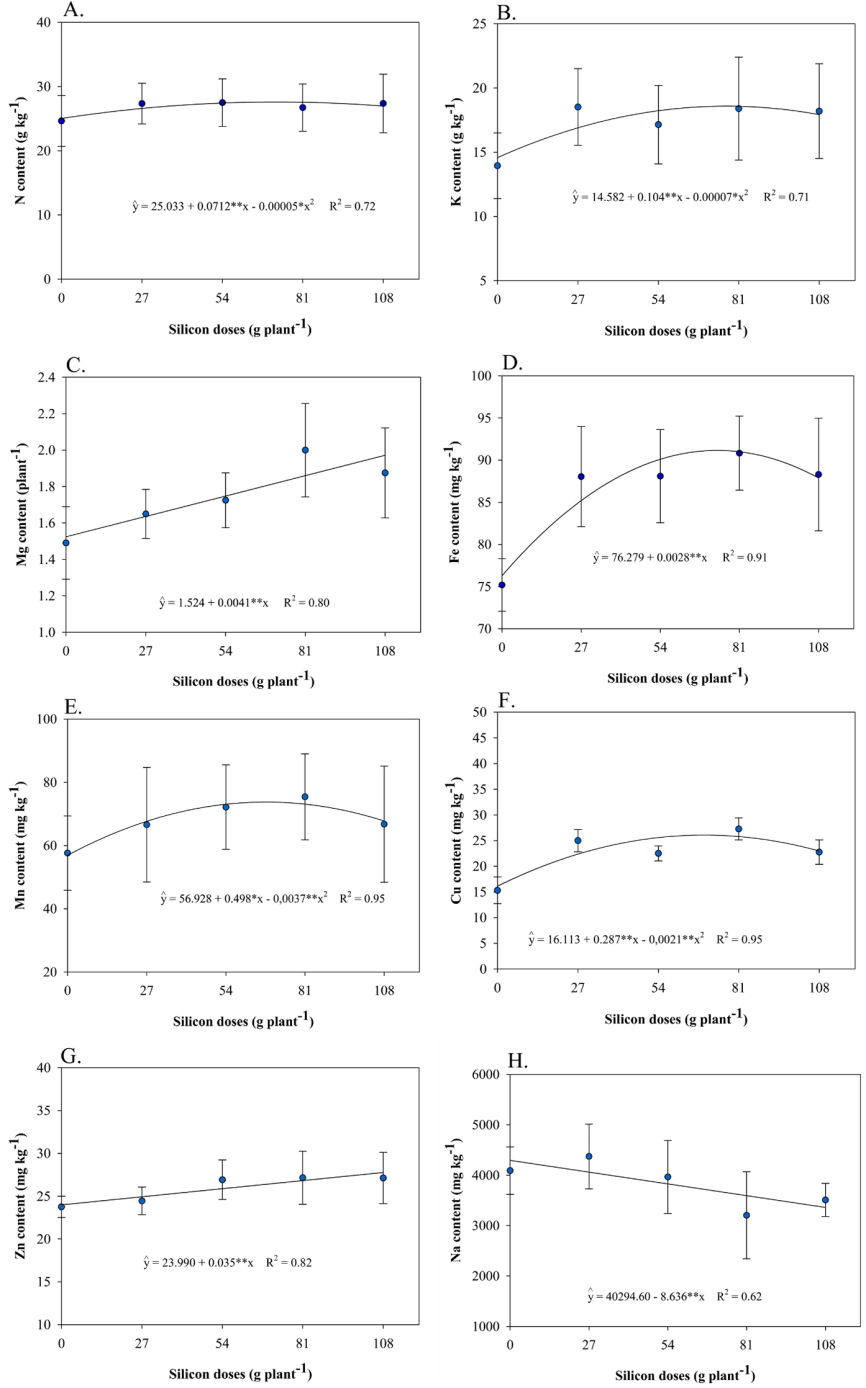
Leaf contents of nitrogen **(A)**, potassium **(B)**, magnesium **(C)**, iron **(D)**, manganese **(E)** and copper **(F)**, zinc **(G)** and sodium **(H)** in sour passion fruit as a function of silicon doses in soil. **,*Values significant at 1% and 5% probability by the F test, respectively. Error bars represent the standard error of the mean (n = 4).

The application of silicon in the soil promoted greater leaf nutrition in sour passion fruit in terms of N, K, Mg, Fe, Mn, Cu and Zn, while reducing the presence of Na in the leaf tissues (**Figure 3**). The highest dose of Si applied increased leaf Mg content from 1.52 to 2.24 g kg^-1^ (**Figure 3C**) and leaf Zn content from 23.99 to 30.29 mg kg^-1^ (**Figure 3G**), while reducing leaf Na content from 4294.60 to 2740.12 mg kg^-1^ (**Figure 3H**), compared to the absence of silicate fertilization. Leaf contents of nitrogen (**Figure 3A**), potassium (**Figure 3B**), iron (**Figure 3D**), manganese (**Figure 3E**) and copper (**Figure 3F**) in sour passion fruit were increased up to the respective Si doses of 71.0 (27.55 g kg^-1^ of N), 74.28 (18.44 g kg^-1^ of K), 72.32 (90.92 mg kg^-1^ of Fe), 67.29 (73.68 mg kg^-1^ of Fe) and 68.33 g plant^-1^ (25.91 mg kg^-1^ of Cu).

**Figure 4:**
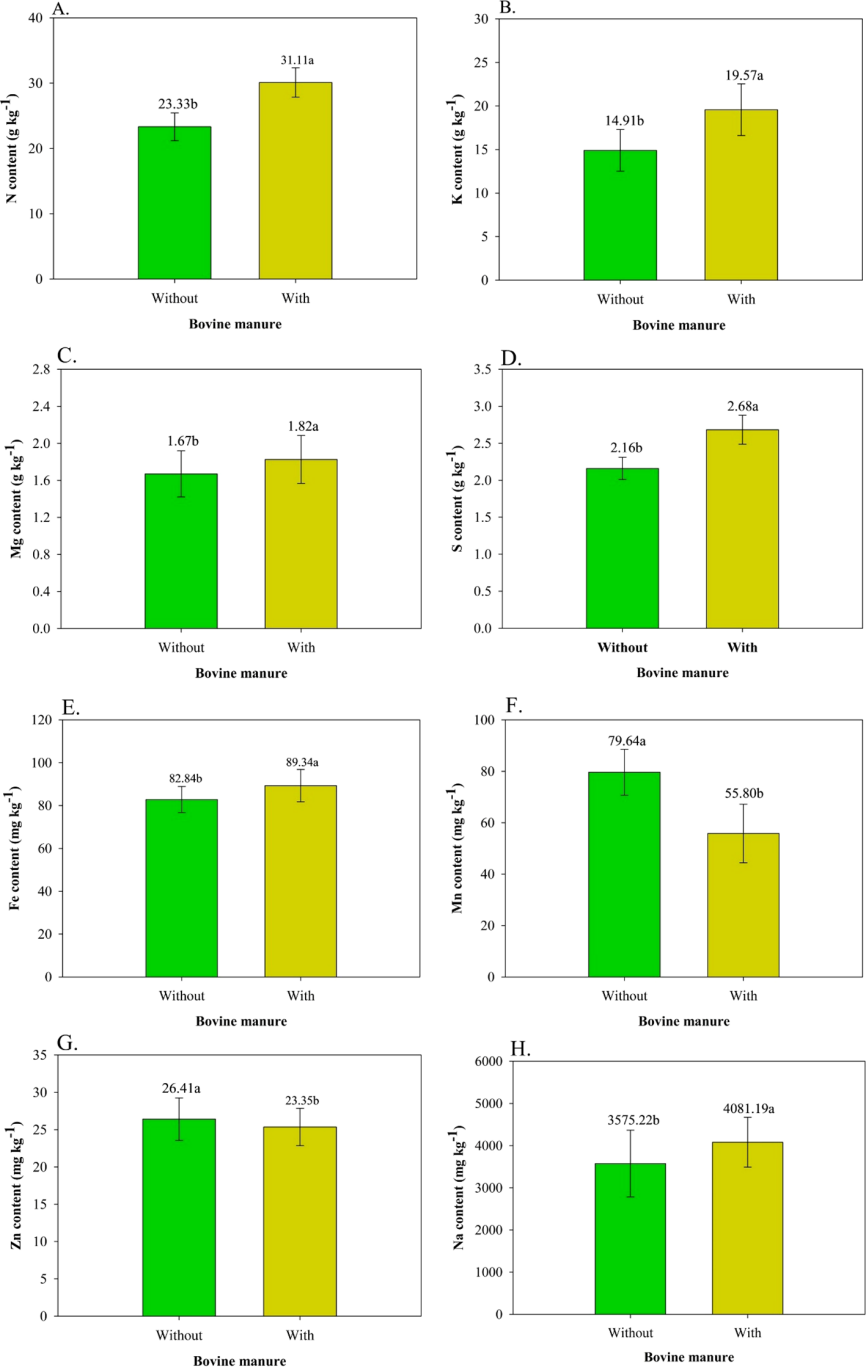
Leaf contents of nitrogen **(A)**, potassium **(B)**, magnesium **(C)**, sulfur **(D)**, iron **(E)** and manganese **(F)**, zinc **(G)** and sodium **(H)** in sour passion fruit as a function of organic matter application in the soil. **Values significant at 1% probability by the F test. Error bars represent the standard error of the mean (n = 4).

The organic matter applied to the soil in the form of cattle manure promoted increments in the leaf contents of N, K, Mg, S, Fe and Na^+^ ion in sour passion fruit, but caused reductions in the leaf contents of Mn and Zn (**Figure 4**). When comparing the presence and absence of manure application, there were increments in the leaf contents of N, K, Mg, S, Fe, Mn, Zn and Na of 33.34%, 24.61%, 9.58%, 31.01%, 42.72% and 14.51%, respectively (**Figures 4A–E, H**) and reductions of 29.9% and 4.01% in the leaf Mn and Zn contents of sour passion fruit (**Figures 4F, G**).

**Figure 5:**
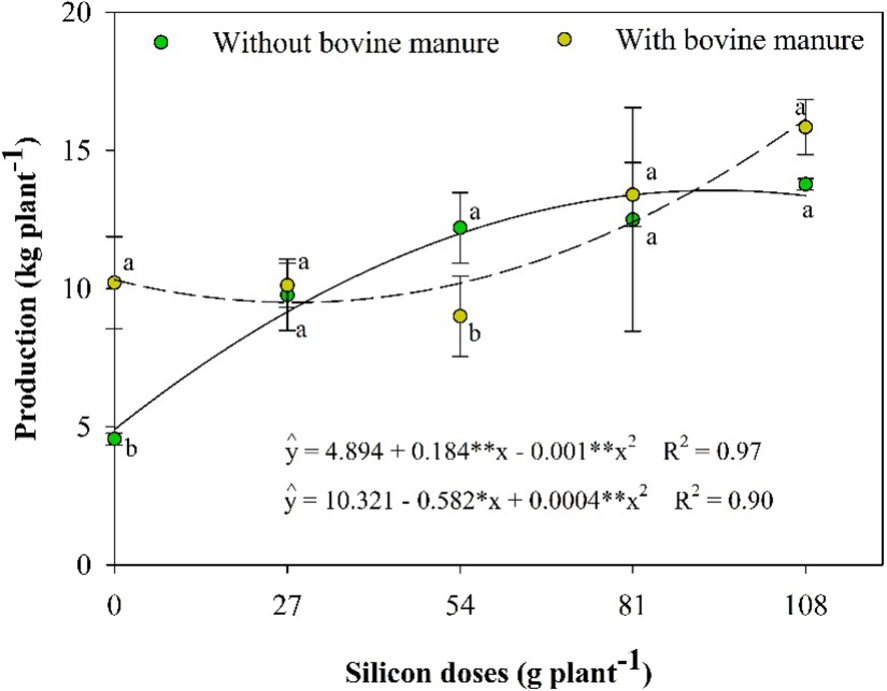
Production of sour passion fruit as a function of silicon doses and without (―) and with (- - -) organic matter in the soil. **,*Values significant at 1% and 5% probability by the F test, respectively. Error bars represent the standard error of the mean (n = 4).

The increase in silicon doses in the soil increased fruit production in sour passion fruit, especially in the soil with organic matter (**Figure 5**). In the soil without organic matter, the production of sour passion fruit was increased up to the dose of 92 g plant^-1^ of silicon (13.35 kg plant^-1^), while in the soil with organic matter, the highest production was obtained with the application of 108 g plant^-1^ of Si (15.69 kg plant^-1^). When comparing the values at the respective doses of 92 and 108 g plant^-1^ of Si, it was observed that the cattle manure promoted increments of 17.5% in sour passion fruit production.

**Figure 6:**
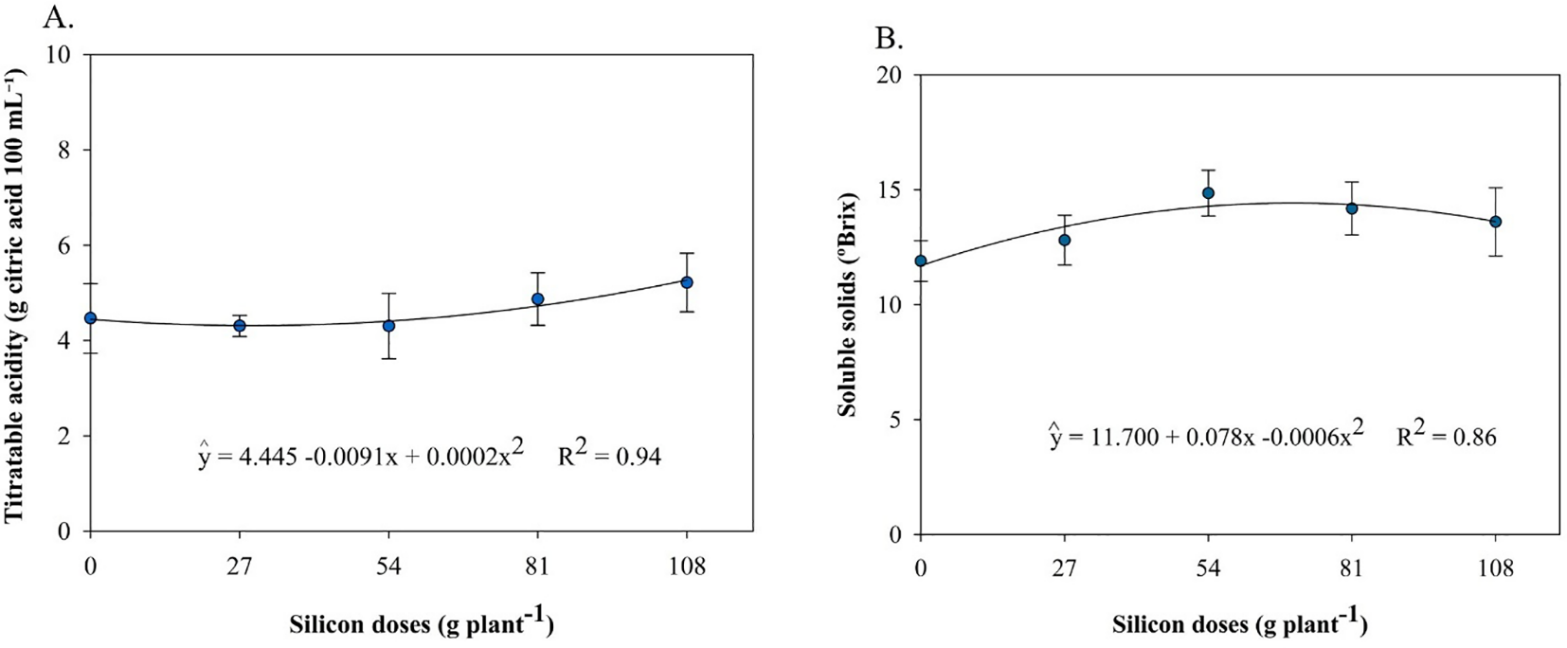
Titratable acidity **(A)** and soluble solids **(B)** of the fruits in sour passion fruit as a function of silicon doses in soil. **,*Values significant at 1% and 5% probability by the F test, respectively. Error bars represent the standard error of the mean (n = 8).

The titratable acidity and soluble solids of passion fruit increased with increasing doses of Si (**Figure 6**). Increasing the Si dose from 0 to 108 g plant^-1^ increased TA by 30.37% (**Figure 6A**), while SS increased up to the estimated maximum Si dose of 65 g plant^-1^, with a value of 14.23°Brix and increases of 21.66% compared to the fruits of plants without silicon application (**Figure 6B**).

**Figure 7:**
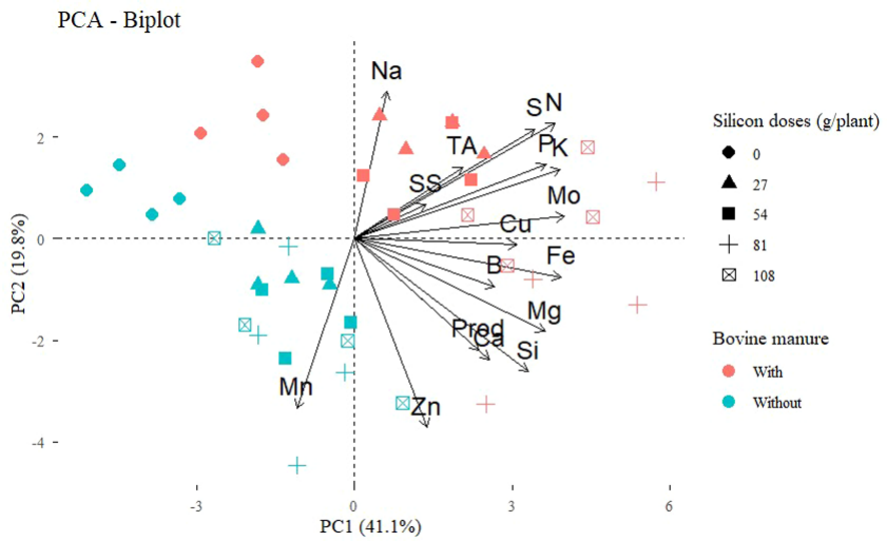
Principal component analysis of nutritional variables and fruit production of sour passion fruit as a function of silicon doses and cattle manure in soil.

As presented in the principal component analysis (PCA) of the leaf mineral composition and fruit production variables of sour passion fruit (**Figure 7**), the eigenvectors of PCA1 and PCA2 were responsible for explaining 41.1% and 19.8% of the variance, respectively, which corresponds to 60.9% of the eigenvectors of the total data variance. PC1 emerges as the most important axis in distinguishing the effects of treatments on the nutritional variables, production, and post-harvest characteristics of the sour passion fruit.

Notably, the elements nitrogen (N), magnesium (Mg), copper (Cu), iron (Fe), sodium (Na), and silicon (Si) show a strong positive correlation with each other, forming a cohesive group on the right side of the biplot (**Figure 7**). This indicates that the application of bovine manure promotes a simultaneous and synergistic increase in nutrient absorption. Furthermore, silicon promotes increased nutrient absorption, especially of N, Cu, and Fe, indicating a beneficial role in plant mineral nutrition. The post-harvest fruit quality variables are also in this same group, demonstrating the importance of adequate nutritional supply for production quality.

Regarding silicon doses, a clear response is observed between lower and higher applied quantities (**Figure 7**). Lower doses (0 and 24 g plant^-1^) do not have as much influence on nutritional status; however, higher doses (81 and 108 g plant^-1^) shift to regions of the nutritional optimization biplot, verifying that there is a dose response threshold for the manifestation of beneficial effects. Higher Si doses (81–108 g plant^-1^) were associated with improved nutritional status, yield, and fruit quality, particularly when combined with cattle manure. Although maximum production was achieved at 108 g plant^-^¹, doses around ~82 g plant^-1^ provided a more balanced response across nutritional, productive, and quality variables, indicating greater agronomic efficiency.

## 4 Discussion

The results of this study demonstrate that silicon and organic matter act both independently and synergistically to improve the nutritional status and yield of sour passion fruit cultivated under sandy soil conditions in a semiarid environment. Under the conditions of this study, the combined use of these inputs altered nutrient dynamics and enhanced fruit production, as also observed by Diniz et al. (2025). This indicates that both mineral and organic components mitigated nutritional limitations inherent to low-CEC soils.

The experimental soil had a sandy texture with very low fertility and limited water-holding capacity, characterized by high sand content, a small clay fraction, low cation exchange capacity, and low organic matter content (**Table 1**). These characteristics favor nutrient losses by leaching and poor retention (Alghamdi et al., 2024; Musei et al., 2024; Holanda et al., 2025). Diniz et al. (2025) corroborated that, under semiarid sandy conditions, increasing soil organic matter and silicon doses improved plant physiological status and yield, explaining the pronounced response to cattle manure and Si observed here.

The combined application of 108 g Si plant^-1^ and cattle manure consistently increased foliar P, Mo, and Si (**Figures 2A, D, E**), indicating a synergistic interaction that enhanced nutrient availability and uptake. Silicon likely reduced P fixation onto Fe and Al oxides, increasing the labile P fraction, while manure supplied organic P and ligands mobilizing micronutrients (Guntzer et al., 2012; Liang et al., 2015). This behavior aligns with the increases of approximately 31% in foliar P and over 100% in foliar Mo observed at 108 g Si plant^-1^ combined with manure.

Dose-response patterns for Ca (**Figure 2B**) and B (**Figure 2C**) indicate that Si modulated cation mobility and apoplastic transport (Cooke et al., 2016; Greger et al., 2018; Khan et al., 2021; Savic et al., 2023). Manure shifted the Si dose for maximum response increased nutrients contents, suggesting that organic matter improved cation exchange capacity and soil buffering, while Si enhanced root functionality and tissue silicification.

Successive applications of organic matter increase foliar Ca and B in sour passion fruit grown in sandy loam soils 14 by improving cation exchange and nutrient retention. Silicon also enhances Ca availability in the soil solution, as shown by Greger et al. (2018), although the response depends on soil type, cultivation system, and species. Organic matter influences B adsorption and desorption (Padbhushan and Kumar, 2017), and B bound to organic surfaces represents the second most available form to plants, explaining the higher foliar B with cattle manure (**Figure 3C**). The B released from organic matter likely acted synergistically with Si, which can stimulate B uptake (Greger et al., 2018).

Independent effects of Si on N (**Figure 3A**), K (**Figure 3B**), Mg (**Figure 3C**), Fe (**Figure 3D**), Mn (**Figure 3E**), Cu (**Figure 3F**), and Zn (**Figure 3G**), along with the reduction in leaf Na (**Figure 3H**), confirm multiple and complementary mechanisms of Si action. At the highest Si dose compared with the lowest, silicon increased leaf Mg by 47% and Zn by 26%, while decreasing Na by 36%, likely through root apoplastic reinforcement and modulation of membrane transporters ( (Coskun et al., 2016; Debona et al., 2017).

Cattle manure improved the nutritional status of sour passion fruit by increasing leaf N, K, Mg, S, Fe, and Na, while slightly reducing Mn and Zn (**Figure 4**). This effect is attributed to the manure’s high organic matter, total carbon, macro- and micronutrient contents, and cation exchange capacity (**Table 2**), which enhance nutrient retention and gradual release (Duangpan et al., 2022). The 33% increase in leaf N reflects continuous mineralization of organic N compounds (Whalen et al., 2000), while the 25% rise in K is linked to the manure’s K supply and CEC contribution. Increases in Mg (10%) and S (31%) further indicate enhanced nutrient cycling and enzymatic activity stimulated by organic inputs (Duangpan et al., 2022).

The improvement in leaf nutrient status was directly associated with increased fruit yield. Combined Si and manure application increased fruit production by approximately 17.5% compared to Si alone (**Figure 5**), indicating that enhanced nutritional balance was a key factor driving yield. Higher foliar P, Ca, K, Mg, B, Mo, Fe, and Si contributed to flowering, fruit filling, and biomass accumulation, while reduced Na favored osmotic adjustment and physiological performance. In sour passion fruit, Silva et al. (2025) reported that application of silicon combined with potassium increased foliar P, K, Ca, Mg, and Fe while reducing Na, indicating that improved nutrient status and lower Na favor growth and biomass accumulation. Although B and Mo were not assessed in that study, our results extend these findings, showing that enhanced foliar B and Mo also improved reproductive performance and physiological efficiency under the studied conditions.

In addition to influencing yield, Si also modified fruit quality, as reflected by the increases in titratable acidity and soluble solids (**Figure 6**). These improvements suggest stimulation of metabolic pathways associated with sugar and organic acid biosynthesis during fruit development. Similar responses have been documented in other fruit crops, where Si enhanced metabolic activity and improved chemical attributes. Yang et al. (2024) reported that Si application increased the sugar-acid balance and elevated key phenolic compounds in tomato fruit, indicating enhanced carbon allocation and secondary metabolism. Likewise, He et al. (2025) demonstrated that Si upregulated genes involved in carotenoid and vitamin C biosynthesis, promoting greater accumulation of these compounds. Together, these findings reinforce that Si functions as a biochemical modulator of fruit quality, supporting the patterns observed in sour passion fruit under the conditions of this study.

The PCA supported the nutritional and productive responses by integrating leaf nutrient composition, fruit production, and post-harvest quality into a single multivariate framework (**Figure 7**). Treatments combining Si and cattle manure formed a distinct cluster, indicating coordinated increases in P, K, Ca, Mg, B, Mo, Cu, Fe, and Si, along with reduced Na levels, patterns strongly associated with higher fruit yield and improved fruit quality attributes. Conversely, treatments without the combined inputs remained close to the origin, demonstrating a restricted multivariate response, with limited variation in nutrient status or fruit production.

This vector arrangement reveals positive correlations among essential nutrients, yield, and fruit quality variables, and a negative correlation with Na, showing that Si and organic matter acted synergistically to optimize ionic balance and overall metabolic efficiency. Physiologically, Si accumulation enhanced cation uptake and fruit production performance while mitigating Na-induced ionic stress. Therefore, the PCA demonstrates that yield gains and post-harvest improvements in sour passion fruit grown in sandy, low-fertility soil resulted from integrated multielement and functional adjustments rather than isolated nutrient effects.

Overall, under the conditions of this study, the combination of soil-applied Si and cattle manure represents an effective agronomic strategy, enhancing nutrient uptake efficiency and ionic regulation, and improving the yield potential of sour passion fruit cultivated in sandy, semiarid soils, as also evidenced by previous studies (Mehrabanjoubani et al., 2015; Greger et al., 2018; Hurtado et al., 2019; Buchelt et al., 2020; Duangpan et al., 2022; Rea et al., 2022; Diniz et al., 2025).

## 5 Conclusions

The combined application of silicon at doses above 81 g plant^-1^ and cattle manure markedly enhanced mineral nutrition and fruit production in sour passion fruit. Silicon increased leaf concentrations of P, Ca, B, Mo, and Si while reducing Na accumulation, whereas cattle manure further elevated leaf N, K, Mg, S, Fe, and Na. In addition, Si also enhanced fruit quality by increasing titratable acidity and soluble solids. While the highest fruit yield was obtained at 108 g plant^-1^ of Si, the dose of 81 g plant^-1^ combined with cattle manure provided the best overall agronomic performance, ensuring a more balanced enhancement of nutrient status, yield, and fruit quality. These findings indicate that the integrated use of Si and organic amendments is an effective agronomic strategy to improve nutrient uptake, ionic balance, and yield of sour passion fruit grown under sandy, semiarid soil conditions.

## Data Availability

The raw data supporting the conclusions of this article will be made available by the authors, without undue reservation.
